# Whole-genome sequencing-based species classification, multilocus sequence typing, and antibiotic resistance mechanisms of the clinical *Aeromonas* complex

**DOI:** 10.3389/fmicb.2025.1473150

**Published:** 2025-02-25

**Authors:** Junwan Lu, Lei Zhang, Chunxia Yan, Naru Lin, Yuan Zhang, Yuning Sha, Jingxuan Zhao, Jun Lu, Qiyu Bao, Guozhi Zhang

**Affiliations:** ^1^Medical Molecular Biology Laboratory, School of Medicine, Jinhua University of Vocational Technology, Jinhua, China; ^2^Department of Clinical Laboratory, Quzhou People’s Hospital/The Quzhou Affiliated Hospital of Wenzhou Medical University, Quzhou, China; ^3^Key Laboratory of Medical Genetics of Zhejiang Province, Key Laboratory of Laboratory Medicine, Ministry of Education, China, School of Laboratory Medicine and Life Sciences, Wenzhou Medical University, Wenzhou, China; ^4^Wenzhou Center for Disease Control and Prevention (Wenzhou Health Supervision Institute), Wenzhou, China

**Keywords:** *Aeromonas* complex, antimicrobial resistance mechanism, whole-genome sequencing, species classification, multilocus sequence typing

## Abstract

**Background:**

Multidrug-resistant strains of the genus *Aeromonas* can produce various β-lactamases that confer resistance to a broad spectrum of β-lactams, which poses a significant public health threat due to their emergence and spread in clinical settings and natural environments. Therefore, a comprehensive investigation into the antibiotic resistance mechanisms of *Aeromonas* is scientifically significant.

**Methods:**

Between 2018 and 2021, 78 clinical *Aeromonas* isolates were collected from human clinical specimens. The MicroScan WalkAway system and average nucleotide identity (ANI) analyses were used to classify the bacterial species. Antibiotic susceptibility was determined through the minimum inhibitory concentration (MIC) test via the agar dilution method. To determine the resistance mechanism and the structure of the resistance gene-related sequences, molecular cloning, whole-genome sequencing and bioinformatic analysis were performed.

**Results:**

Among the 78 *Aeromonas* isolates studied in this work, obtained from various specimens from different clinical departments, 77 were classified into seven known species by ANI analysis. Most of the isolates were *A. caviae* (34.6%, 27/78), followed by *A. hydrophila* (25.6%, 20/78). Multilocus sequence typing (MLST) revealed that they belonged to 72 sequence types (STs), including 52 new STs. A total of 334 resistance genes of 30 antibiotic resistance genotypes were identified from the genomes, more than half (55.99%, 187/334) of which were β-lactamase genes. The isolates showed much higher rates of resistance to penicillins (penicillin G, 98.7%) and first-generation cephalosporins (cefazolin, 96.2%), but lower resistance rates to fourth-generation cephalosporins (cefepime, 6.4%), monobactams (aztreonam, 5.1%), and carbapenems (imipenem, 1.3% and meropenem, 5.1%). Structural analyses of some β-lactamase genes (such as *bla*_NDM-1_ and *bla*_PER-3_) related sequences revealed that they were generally associated with mobile genetic elements.

**Conclusion:**

The investigation of the correlation between the distribution of β-lactamase genes and *Aeromonas* resistance phenotypes in this study suggested an urgent need for rigorous monitoring and control to counteract the escalating public health threat posed by the increase in *Aeromonas* strains harboring extended-spectrum β-lactamase and metallo-β-lactamase genes.

## Introduction

The genus *Aeromonas* constitutes a ubiquitous group of Gram-negative rods found worldwide in natural environments, particularly in aquatic habitats (Parker and Shaw, 2011). The classification of *Aeromonas* species is complex, and accurate laboratory identification remains a significant challenge. Traditional biochemical tests, 16S rRNA gene homology analysis and matrix-assisted laser desorption/ionization time-of-flight mass spectrometry (MALDI-TOF MS) are unreliable for identifying *Aeromonas* species (Pérez-Sancho et al., 2018). For example, *Aeromonas dhakensis* (previously known as *Aeromonas aquariorum*) is frequently misidentified as *A. hydrophila* by conventional biochemical methods. Accurate identification can be achieved through core genome phylogenetic analysis (Chen et al., 2014a).

*Aeromonas* species produce a variety of virulence factors, including adhesins, cytotoxins, hemolysins, lipases, and proteases; exhibit biofilm formation capacity; use specific metabolic pathways; and exhibit virulence factor expression mediated through quorum sensing (Janda and Abbott, 2010). The pathogenicity of *Aeromonas* appears to differ at the species level. The role of *Aeromonas* species as human pathogens has been highlighted by their occurrence after natural disasters, such as the 2004 tsunami in Thailand, where they were identified as the most common pathogens causing skin or soft tissue infections (Hiransuthikul et al., 2005). *Aeromonas* species can cause a variety of infections in humans, ranging from common conditions such as gastroenteritis to more serious illnesses such as septicemia, peritonitis, cholangitis, and catheter-related infections (Janda and Abbott, 2010; Wu et al., 2007). Both immunocompromised and immunocompetent individuals can be infected with *Aeromonas*, usually via ingestion of or direct mucocutaneous contact with contaminated water or food (Janda and Abbott, 2010). The reported mortality rates of patients with *Aeromonas* bacteremia range from 24 to 63% (Chen et al., 2014b). Three main *Aeromonas* species are associated with human disease: *A. hydrophila*, *A. veronii*, and *A. caviae*. While many *Aeromonas* infections are self-limiting, invasive infections can progress rapidly and become life-threatening for those with underlying conditions or immune system impairments (Zhang et al., 2023; Zhou et al., 2019). In recent years, the widespread presence of *Aeromonas* species such as *A. dhakensis* has been identified to be associated with human infections, and these species may be more deadly than other *Aeromonas* species (Igbinosa et al., 2012).

Furthermore, with the overuse of antibiotics in agriculture, aquaculture, and clinical settings, *Aeromonas* resistance to antimicrobial agents continues to increase. Antibiotic susceptibility varies depending on the geographical region and the *Aeromonas* species tested. Appropriate antimicrobial therapy is necessary for controlling the development of infection. The incidence and severity of *Aeromonas* infections appear to be underestimated, and underreporting remains prevalent in many countries (Kosikowska et al., 2022).

This study aimed to investigate the molecular epidemiology of *Aeromonas* species from a hospital in Quzhou, which is located in southeastern China and has a subtropical climate, over a four-year period. The clinical features, virulence and antimicrobial resistance mechanisms of the clinical *Aeromonas* strains were analyzed, and the findings will advance our understanding of *Aeromonas* infections and help establish appropriate treatment strategies.

## Materials and methods

### Clinical strain collection and species classification

A total of 78 clinical *Aeromonas* isolates were collected from Quzhou People’s Hospital in Zhejiang Province, China, between 2018 and 2021. They were isolated from different sources: excrement (*n* = 35), fester (*n* = 14), bile (*n* = 12), whole blood (*n* = 6), urine (*n* = 6), sputum (*n* = 4), and catheter (*n* = 1). The age of the patients ranged between 9 months and 93 years with an average age 54.8 years. 70.5% (55/78) were from male patients (with an average age of 56.4 years) and 29.5% (23/78) were from female patients (with an average age of 50.5 years). The isolation of *Aeromonas* species was performed by directly streaking the fecal specimen onto the starch ampicillin agar (SAA) plate supplemented with 10 mg/L ampicillin (Solarbio, Beijing, China) and incubated at 37°C for 24 h. For the other specimens, enrichment culture was performed over night in tryptic soy broth (TSB, Solarbio, Beijing, China) and the culture was then streaked onto the SAA plates. Typical yellow colonies indicative of *Aeromonas* species were selected for further identification. Oxidase test was performed using the DrySlide Oxidase kit (BD, Oslo, Norway). The tryptic soy agar (TSA, Solarbio, Beijing, China) plates were used to culture the *Aeromonas* isolates and the bacteria were then preserved in TSB containing 20% glycerol at −80°C for subsequent analysis. Preliminary species identification of these isolates was performed with a MicroScan WalkAway^®^, an automated bacterial identification platform (Siemens AG FWB, Germany) (Khan et al., 2015). All 78 isolates were classified as *Aeromonas* complex isolates. Species/subspecies identification of the *Aeromonas* complex was further performed by ANI analysis using FastANI v1.31, and a value >95% was used as the threshold for species definition (Jain et al., 2018).

### Antimicrobial susceptibility test

According to the previous publication (Weinstein and Lewis, 2020), the breakpoint criteria of the Clinical and Laboratory Standards Institute (CLSI M100) for Enterobacteriaceae were used for penicillin G, cefazolin, and cefoxitin. Antimicrobial susceptibility was determined using the agar dilution method on Mueller–Hinton (MH) agar plates supplemented with different concentrations of antibiotics ranging from 0.004 to 2,048 μg/mL (Table 1). The test was repeated three times to ensure accuracy. The minimum inhibitory concentrations (MICs) were interpreted following the recommended breakpoint criteria for the *Aeromonas hydrophila* complex applicable to other non-Enterobacterales, as outlined by the CLSI (2021). For cefotaxime, ceftriaxone, cefepime, aztreonam, imipenem, and meropenem, the MIC breakpoints for other non-Enterobacterales were applied.

**Table 1:** Susceptibility profiles and MICs for 78 *Aeromonas* strains.

Antimicrobial agent	CLSI break point interpretation (%)			MIC_50_ (mg/L)	MIC range (mg/L)
	Susceptible	Intermediate	Resistant		
Penicillin G	1.3	0	98.7	2,048	4–2,048
Cefazolin	1.3	2.6	96.2	128	0.25–1,024
Cefoxitin	47.4	16.7	35.9	80	1–512
Cefotaxime	85.9	0	14.1	0.19	0.015–16
Ceftriaxone	85.9	0	14.1	0.25	0.015–128
Cefepime	91.0	2.6	6.4	0.125	0.008–16
Aztreonam	94.9	0	5.1	0.06	0.015–16
Imipenem	94.9	3.8	1.3	0.5	0.03–64
Meropenem	94.9	0	5.1	0.03	0.004–32

### Whole-genome sequencing and sequence analysis

The bacterial genomic DNA was extracted by using an AxyPrep Bacterial Genomic DNA Miniprep Kit (Axygen Scientific, Union City, CA, United States). Genome sequencing was performed by the Illumina HiSeq 2500 and PacBio Sequel IIe platforms by Shanghai Personal Biotechnology Co., Ltd. (Shanghai, China). A library with an average insert size of 400 bp was prepared using the NEBNext Ultra II DNA Library Preparation Kit for Illumina HiSeq 2500 sequencing (paired-end run; 2 × 150 bp). Sequence assembly was conducted *de novo* for Illumina short reads using SPAdes v.3.14.0 (Bankevich et al., 2012). The genomes of isolates carrying metallo-β-lactamase (MBL) genes were further sequenced by PacBio RS II instruments (Pacific Biosciences, CA, United States), and the long reads from PacBio sequencing were assembled by HIFIasm using the data obtained from Illumina sequencing as reference input (Cheng et al., 2021). ORFs present in the genome sequence were predicted by Prokka v1.14.6 (Seemann, 2014), while BLAST analysis against the NCBI protein sequence database helped annotate their function with an e-value threshold of 1e-5. Resistance Gene Identifier v5.2.0 (Jia et al., 2017) and the comprehensive antibiotic resistance database (CARD, McArthur et al., 2013) were used to identify antimicrobial resistance genes. MLST analysis was performed using the database,^1^ with the MLST scheme developed by Martino et al. (2011). This scheme is based on six loci including *gltA*, *groL*, *gyrB*, *metG*, *ppsA* and *recA*. Gene distribution was visualized using the ComplexHeatmap package in R (Gu et al., 2016). The single-copy core gene phylogenetic tree was generated using Roray v3.11.2 (Page et al., 2015) and then visualized with ggtree v3.2.0 (Yu et al., 2018).

### Time-kill assay

According to the minimal inhibitory concentration (MIC) of meropene, piperacillin + tazobactam and ceftazidime to *A. caviae* QZ63 or *A. hydrophila* QZ124, the bactericidal effects of meropene, piperacillin + tazobactam, ceftazidime at various concentrations of 0.5 ×, 1 ×, 2 × MIC were studied by time-kill assay. Following the methods recommended in the previous publicatins (Norden et al., 1979; Paevskiĭ, 1993), two isolates *A. caviae* QZ63 with *bla*_NDM-1_ and *A. hydrophila* QZ124 with *bla*_PER-3_ were selected as test strains, and two isolates *A. caviae* QZ54 free of *bla*_NDM-1_ and *A. hydrophila* QZ87 without *bla*_PER-3_ were used as control strains to study the synergistic bactericidal effects. The experiment procedure is briefly described as follows: cation-adjusted Mueller–Hinton broth (CAMHB) containing 1 × 10^5^ CFU/mL bacteria is mixed with single or combined antimicrobial agents incubated overnight with consecutive shacking at 37°C. Meanwhile, the same broth without antibiotics was served as a control. Broth samples were serially sampled at times of 0, 1, 2, 4, 6, 8, 12 and 24 h and plated onto a Mueller–Hinton plate, respectively. After overnight incubation at 37°C, the colonies were counted.

## Results and discussion

### The months with the more *Aeromonas* isolates

**Figure 1:**
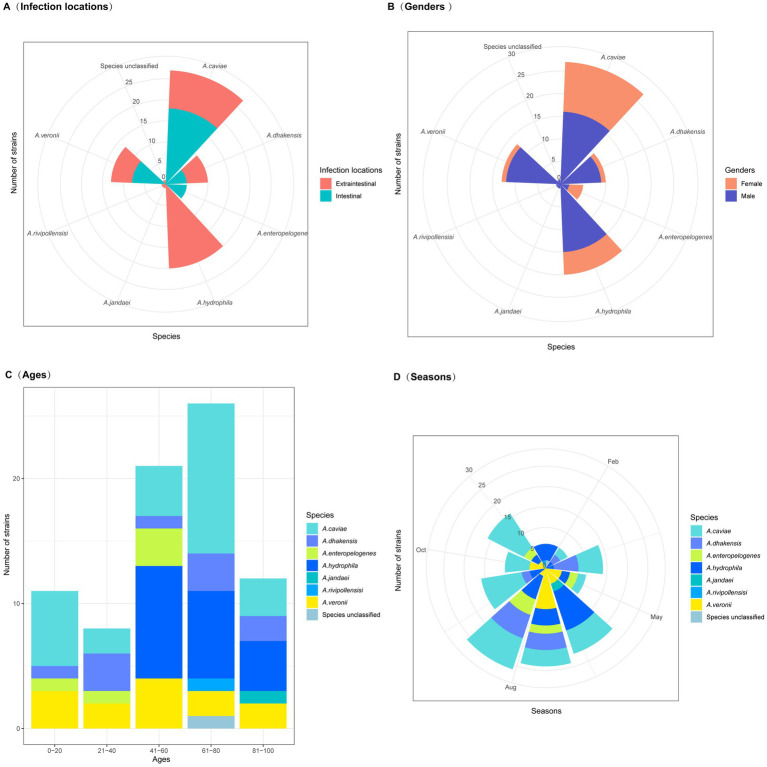
Clinical characteristics of patients with *Aeromonas* infections. **(A)** Infection locations (extraintestinal and intestinal infections). **(B)** Patient gender. **(C)** Patient ages. **(D)** Seasons in which the isolates were obtained.

June, July, and August were the months with the highest incidence rates, 46.2% (36/78) of the isolates were obtained in these 3 months. It was possibly due to favorable environmental conditions for the proliferation and growth of *Aeromonas* during this period of the year (Figure 1). Consequently, this led to an increase in *Aeromonas* levels in the environment and, thus, a greater risk of infection (Janda and Abbott, 2010). *Aeromonas* species are widely distributed aquatic Gram-negative bacteria found in natural environments worldwide. They have become the third most common enteric bacterial pathogens, following the genera *Campylobacter* and *Salmonella* (Yuwono et al., 2021). In recent years, more bacteria of the genus *Aeromonas* than those of the other genera causing diarrhea have been isolated from diarrhea specimens in the outpatient department of Quzhou People’s Hospital, Quzhou, China.

### Species identification and multilocus sequence typing of the isolates

**Figure 2:**
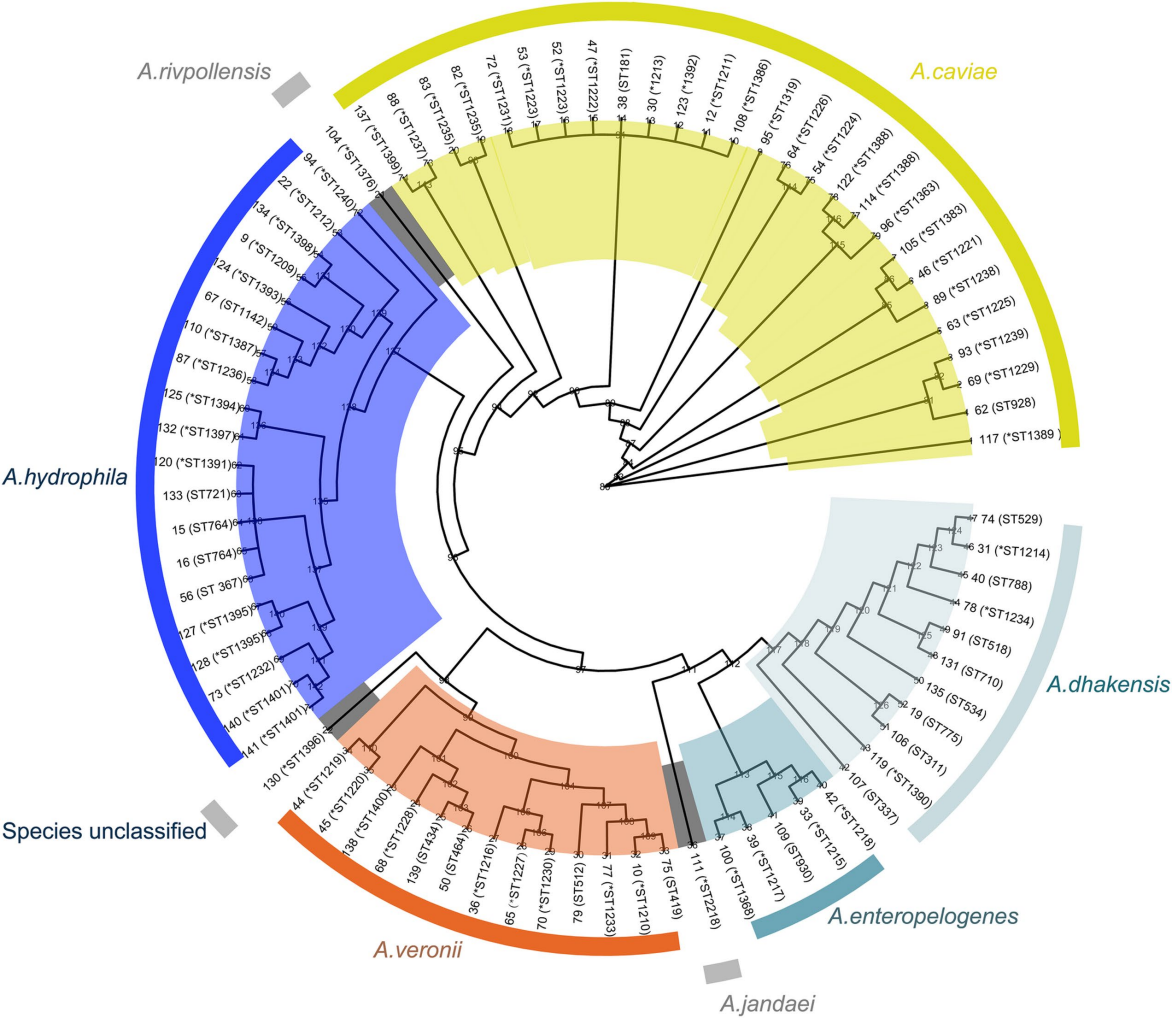
Phylogenetic tree of the genomes of 78 *Aeromonas* complex isolates. The species and sequence types (STs) are depicted in concentric circles, with “*” representing new STs. The numbers on the branches indicate the bootstrap values.

The ANI analysis result of the 78 isolates revealed that the predominant species was *A. caviae* (*n* = 27), followed by *A. hydrophila* (*n* = 20), *A. veronii* (*n* = 13), *A. dhakensis* (*n* = 10), *A. enteropelogenes* (*n* = 5), *A. rivipollensisi* (*n* = 1), *A. jandaei* (*n* = 1) and an unclassified isolate temporarily designated *Aeromonas* sp. 130, which showed the highest ANI of 94.63% with *A. veronii* CIP 107763 (GenBank Accession No. GCF_000820285.1) (according to the criteria of the ANI analysis, an isolate that showed an ANI below the threshold of 95% for any classified species was considered a novel species) (Figure 2). Notably, there were discrepancies between the identification results from the ANI analysis and the MicroScan WalkAway system (MSWS). Some isolates identified as *A. caviae* by ANI were *A. enteropelogenes* or *A. hydrophila* by MSWS. Similar results occured in other species. Several isolates of *A. hydrophila*, *A. veronii* and *A. rivpollensi* by ANI were identified as *A. veronii*, *A. hydrophila*, and *A. veronii* by MSWS, respectively. Furthermore, since the MSWS database does not contain *A. dhakensis* data, the strains of the species *A. dhakensis* identified by the ANI analysis were previously identified as *A. hydrophila*, *A. jandaei* or *A. caviae* by MSWS (Supplementary Table S1). It has been recognized that bacterial species classification by ANI analysis was a gold standard (Jain et al., 2018). Difference did exist between species identification results by ANI and by other methods such as traditional biochemical tests, commercial identification kits, or automatic or semiautomatic systems, which might lead confusion of different species of the genus or misidentification of one species as a member of other genera (Deng et al., 2014; Janda and Abbott, 2010). The whole-genome sequencing-based method exhibited relatively high accuracy in identifying bacterial species (Beaz-Hidalgo et al., 2010; Martínez-Murcia et al., 2005). In this work, the overall concordance rate between the ANI and MSWS analyses for the classification of all *Aeromonas* species was 71.79%. If the *A. dhakensis* isolates were excluded because there is no information for this species available in the MSWS database, the concordance rate between the two methods reached 83.58%.

Among the 35 *Aeromonas* isolates that cause intraintestinal infections, *A. caviae* accounted for the greatest proportion (51.43%, 18/35), followed by *A. veronii* (20.0%, 7/35), *A. enteropelogenes* (14.29%, 5/35), and *A. dhakensis* (14.29%, 5/35). In contrast, among the *Aeromonas* isolates that cause extraintestinal infections, *A. hydrophila* accounted for the largest proportion (46.51%, 20/43), followed by *A. caviae* (20.93%, 9/43), *A. dhakensis* (13.95%, 6/43), *A. veronii* (11.63%, 5/43), *A. jandaei* (2.33%, 1/43), *A. rivipollensisi* (2.33%, 1/43), and the unclassified *Aeromonas* sp. isolate 130 (2.33%, 1/43) (Figure 2). Although *Aeromonas* species primarily cause intraintestinal infections (Zhou et al., 2019), there have been an increasing number of reports in recent years about extraintestinal infections caused by *Aeromonas* (Sinclair et al., 2016). Similar to the results of this study, extraintestinal infections were found to be caused mainly by *A. hydrophila* (Chen et al., 2021), while intraintestinal infections were caused primarily by *A. caviae* (Zhou et al., 2019).

These 78 *Aeromonas* isolates exhibited diverse sequence types (STs) according to multilocus sequence typing (MLST), and 72 STs were identified, including 53 novel STs (ST1209-1240, ST1319, ST1363, ST1368, ST1383 and ST1386-1401) (Supplementary Table S1). Among these STs, new STs were predominant, accounting for 73.61% (53/72). Most STs (91.67%, 66/72) had only one isolate each, and the six STs with more than one isolate were ST1388 (*n* = 3), ST1223 (*n* = 2), ST1235 (*n* = 2), ST1401 (*n* = 2), ST764 (*n* = 2) and ST311 (*n* = 2) (Supplementary Table S1).

### The resistance phenotypes of *Aeromonas*

Among the nine β-lactams tested, most isolates were resistant to penicillin G (98.7%, 77/78) and the first-generation cephalosporin cefazolin (96.2%, 75/78). A total of 35.9% (28/78) of the isolates were resistant to the second-generation cephalosporin cefoxitin. A total of 14.1% (11/78) of the isolates were resistant to both the third-generation cephalosporins, cefotaxime and ceftriaxone. High susceptibility to fourth-generation antibiotics, namely, a cephalosporin (cefepime), a monobactam (aztreonam), and two carbapenems (imipenem and meropenem), was detected, with resistance rates of 6.4% (5/78), 5.1% (4/78), 1.3% (1/78) and 5.1% (4/78), respectively (Table 1). Only 7.69% (6/78) of the *Aeromonas* strains showed resistance to one or more of the four antimicrobial agents cefepime, aztreonam, imipenem or meropenem, and uncommon β-lactamase genes were present in these strains. Among the four imipenem-resistant strains, three *A. cavese* strains (isolates QZ63, QZ114, QZ122) carried *bla*_NDM-1,_ and one *A. cavese* strain (isolate QZ62) carried *bla*_IMP-26_. The other two strains (*A. cavese* QZ117 and *A. hydrophila* QZ124) carrying *bla*_PER-3_ showed resistance to cefepime and/or aztreonam. Except for five isolates of the species *A. enteropelogenes* with one *bla*_TRU_ gene each, the other (73) isolates generally encoded two or more genotypes of the β-lactamase genes (Table 1; Supplementary Table S3).

### Distribution of resistance genes among *Aeromonas* isolates

**Figure 3:**
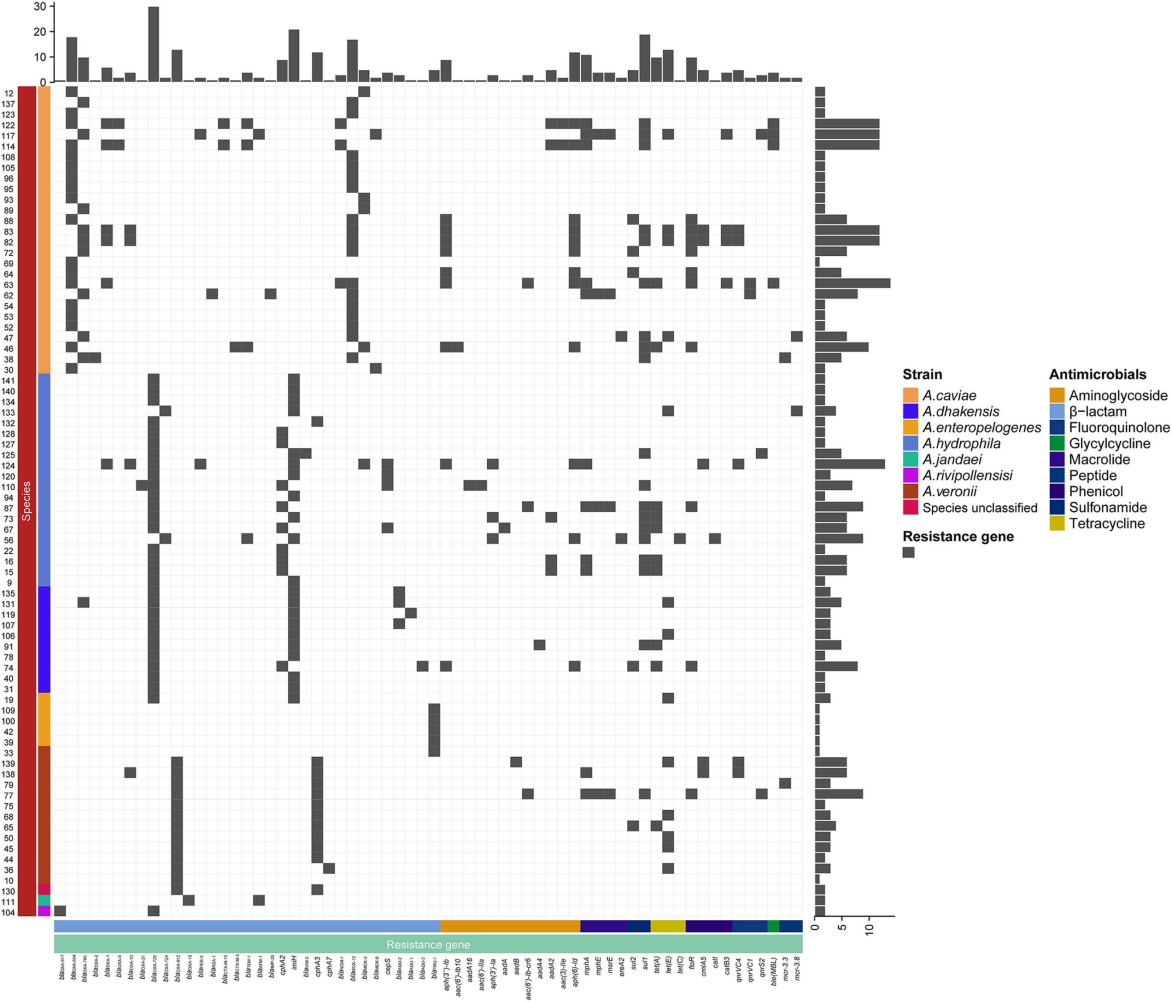
Distributions of the β-lactamase genes in the 78 isolates. The blue and white squares represent the presence and absence of the β-lactamase gene, respectively.

A total of 334 resistance genes (>80% aa similarity with the functionally characterized resistance genes) of 30 genotypes (with 65 subgenotypes) associated with eight antimicrobial agent categories were identified in the genomes of the 78 *Aeromonas* isolates. The category with the most genes (or genotypes) was β-lactams. More than a half (55.99%, 187/334) of the resistance genes were β-lactamase genes from 15 genotypes, including *bla*_PER_, *bla*_RSA_, *bla*_CTX-M_, *bla*_TEM_, *bla*_cphA_, *bla*_IMP_, *bla*_AFM_, *bla*_imiH_, *bla*_NDM_, *bla*_VIM_, *bla*_MOX_, *bla*_CEPS_, *bla*_AQU_, *bla*_TRU_, and *bla*_OXA_. Seven genotypes of aminoglycoside genes [*aph(3″)*, *aph(3′)*, *aph(6)*, *aac(6′)*, *aac(3)*, *aadA*, and *ant(2″)*] (11.97%, 40/334), and three genotypes of phenicol resistance genes (*floR*, *cmlA*, and *cat*) were found, while for the remaining five antimicrobial categories, only one genotype each was present, which included fluoroquinolone (*qnr*), tetracycline (*tet*), sulfonamide (*sul*), peptide (*mcr*) and glycylcycline (*ble*_MBL_) resistance genes (Figure 3).

**Figure 4:**
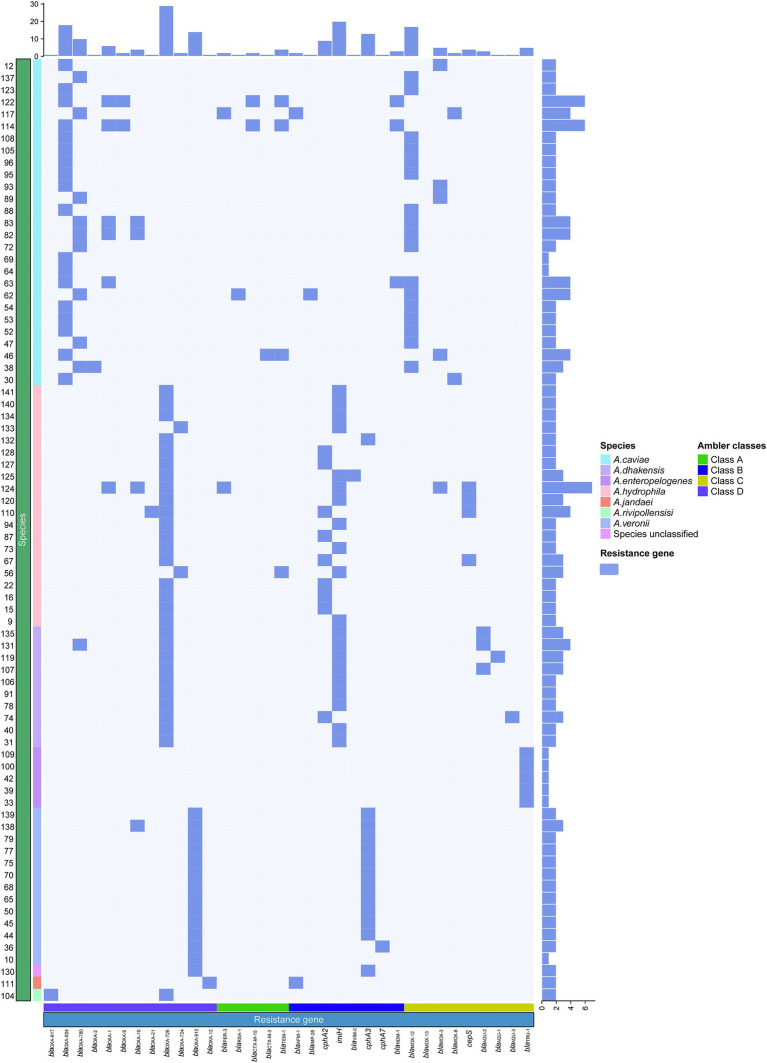
Synteny analysis of the 31.7 kbp MDR region in the chromosome of *A. caviae* QZ63. hp, hypothetical protein. Accession numbers: *Enterobacter hormaechei* 22KM1314 chromosome (CP126807.1), *Enterobacter hormaechei* 22KM0858 chromosome (CP126811.1), plasmid pS44-1 of *Aeromonas salmonicida* S44 (CP102110.1), *Aeromonas caviae* GD21SC2284TT chromosome (AP021927.1), *Aeromonas hydrophila* WP2-W18-ESBL chromosome (CP102110.1), plasmid P2 of *Klebsiella pneumoniae* 1,083 (OW967807.1), and plasmid pLZ135-NDM from an *Escherichia coli* isolate (MF353156.1). *orfA*, *orfB*: two fusion protein formed by translational frame-shifting which were the transposase for IS*1133*. *orf*: DUF3363 domain-containing protein.

As β-lactams are the most important antimicrobials for treating bacterial infectious diseases, this study focused mainly on β-lactam resistance mechanisms. As mentioned above, of the 334 resistance genes identified in the 78 genomes, more than a half (55.99%, 187/334) were β-lactamase genes of 15 genotypes (34 subgenotypes), which covered all four classes [A (5.3%, 10/187), B (26.7%, 50/187), C (20.4%, 38/187), D (89/187, 47.59%)] (Figure 4). According to the Ambler (1980) classification system, among the last four genotypes, except for *bla*_VIM_, which is found in *A. hydrophila*, the other three are all found in *A. caviae*. Class C contained four genotypes, namely, *bla*_MOX_ [*n* = 26, with 96.2% (25/26) in *A. caviae* and 3.8% (1/26) in *A. hydrophila*], *bla*_CEPS_ (*n* = 4), *bla*_AQU_ (*n* = 5), and *bla*_TRU_ (*n* = 5), with the last three being uniquely identified in *A. hydrophila*, *A. dhakensis*, and *A. enteropelogenes*, respectively. However, class D had only one genotype, *bla*_OXA_ (*n* = 89) (Supplementary Table S2). Notably, the class D β-lactamase gene *bla*_OXA_ was the most prevalent (89/187, 47.59%) and was identified in all 78 *Aeromonas* isolates, except for five isolates of the species *A. enteropelogenes*. *A. cavese* and *A. hydrophila* had nine and eight genotypes, respectively, from all four classes of Ambler β-lactamase genes. In *A. dhakensis*, four genotypes of three classes (B, C, and D) were present. In the *A. veronii* isolates, *A. jandaei* 111 and *Aeromonas* sp. 130, two genotypes (*bla*_cphA3_ of class B and *bla*_OXA_ of class D) were found, while in the *A. enteropelogenes* isolates and *A. rivipollensisi* 104, only *bla*_TRU_ of class C and *bla*_OXA-912_ of class D were identified. Among the 15 genotypes, nine were present in only one species. Interestingly, the species *A. enteropelogenes* (previously known as *A. tructior*/*A. trota*) consistently demonstrated susceptibility to ampicillin and is also the only known *Aeromonas* species that produces the single class C β-lactamase *bla*_TRU-1_ (Chen et al., 2012). This finding aligns with the result observed in this study.

### Comparative analysis of the multiresistance region in *Aeromonas caviae* QZ63 carrying two copies of the metallo-β-lactamase gene *bla*_NDM-1_

To analyze the molecular mechanism underlying aztreonam and carbapenem resistance, the complete genomes of two isolates, *A. hydrophila* QZ124 (resistant to imipenem), encoding a *bla*_NDM-1_ gene, and *A. caviae* QZ63 (resistant to meropenem and cefepime), encoding a *bla*_PER-3_ gene, were sequenced. *A. hydrophila* QZ124 had a plasmid designated p124-100, however *A. caviae* QZ63 was free of a plasmid. The *A. caviae* QZ63 and *A. hydrophila* QZ124 genomes encoded 18 (13 genotypes) and 23 resistance genes (16 genotypes), respectively (Tables 2, 3), which showed ≥95% similarity to the functionally characterized resistance genes in the CARD. Interestingly, 83.33% (15/18) of the resistance genes of *A. caviae* QZ63, including three β-lactamase genes (two *bla*_NDM-1_ genes and one *bla*_OXA-1_ gene), were clustered in an approximately 31.7 kb multidrug resistance (MDR) region.

**Table 2:** Resistance genes identified in the *A. caviae* QZ63 genome.

Antimicrobial class	Resistance gene
β-lactam	*bla*_OXA-504_ *bla*_OXA-1_ *bla*_MOX-12_ *bla*_NDM-1_x2
Aminoglycoside	*aac(6′)-Ib-cr6* *aph(3″)-Ib* *aph(6)-Id*
Bleomycin	*ble*_MBL_x2
Sulfonamide	*sul1*x2
Macrolide	*mphA*
Chloramphenicol	*catB3*
	*floR*
Tetracycline	*tet(A)* *tet(R)*
Quinolone resistance	*qnrVC1*

**Table 3:** Resistance genes identified in the *A. hydrophila* QZ124 genome.

Antimicrobial class	Chromosome	p124-100
β-lactam	*bla*_PER-3_	*bla*_MOX-3_
	*bla*_OXA-726_	*bla*_OXA-1_
	*cepS*	*bla*_OXA-10_
	*cphA2*	
Aminoglycoside		*aac(6′)-Ib4*
		*aac(6′)-Ib-cr6*
		*aac(6′)-Ib9*
		*ant(3″)-IIa*
Sulfonamide	*sul1x2*	*sul1*
Macrolide	*mphA*	*mphA*
Chloramphenicol		*catB3* *cmlA5*
Quinolone		*qnrVC4*
Rifampin		*arr-3*
Quaternary ammonium compound		*qacEΔ1*
Posfomycin		*fosC2*
Trimethoprim		*dfrA14*

**Figure 5:**
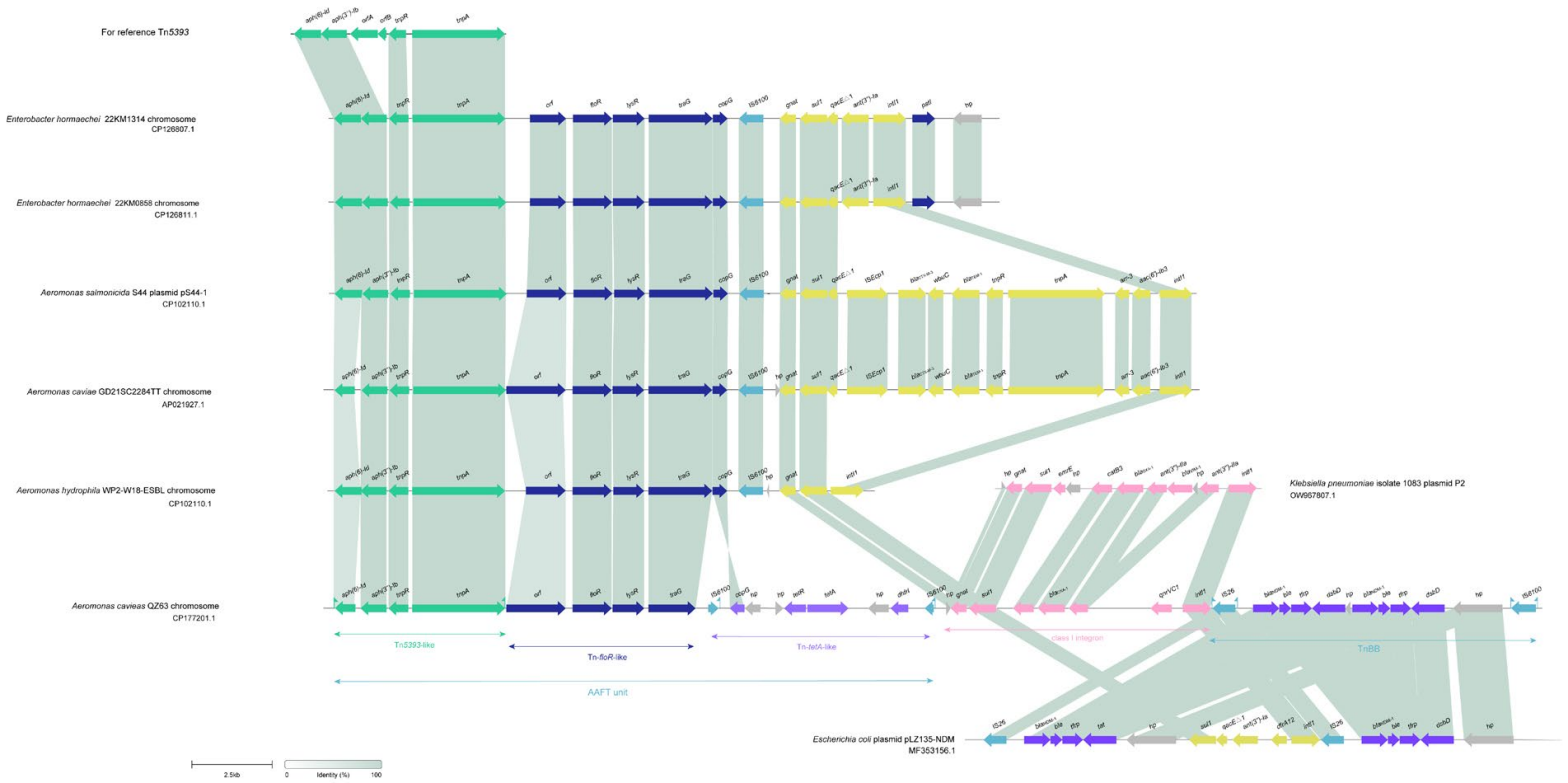
Distributions of resistance genes in the 78 isolates. The blue and white squares represent the presence and absence of the resistance gene, respectively. The categories of resistance genes and the species of the isolates are marked with different colors.

The analysis of this MDR region of *A. caviae* QZ63 revealed that it consisted of three main parts encoding resistance genes associated with the mobile genetic elements (MGEs): an unit (named AAFT unit) encoding four resistance genes *aph(6)-Id*, *aph(3″)-Ib, floR* and *tet(A)*, a class I integron (*hp-gnat-sul1-catB3-bla_OXA-1-_ant(3″)-IIa-qnrVC1-intl1*), and a transposon IS*26*-*bla*_NDM-1_-*ble*-*tfrp*-*dsbD*-*hp*-*bla*_NDM-1_*-ble*-*tfrp*-*dsbD*-*hp*-IS*6100* (TnBB). Further analysis of the structure of the AAFT unit revealed that it was composed mainly of three MGEs or MGE-like fragments, which included an *aph(6)-Id* and *aph(3″)-Ib*-encoding Tn*5393*-like fragment, a *floR*-encoding fragment (*floR*-fragment) and a *tetA* and *dhfr1*-encoding transposon-like fragment (Tn-*tetA*-like). When using the 31.7 kb-MDR region as a query to search for similar sequences in the NCBI nonredundant nucleotide database, five sequences carrying two fragments (Tn*5393*-like and *floR*-fragment) of the AAFT unit were retrieved (Figure 5). These sequences included one from the same species as in this work (GenBank Accession No. AP021927.1, *Aeromonas caviae* str. WP2-W18-ESBL-01, isolated from wastewater treatment plant effluent in Japan, with a coverage of 75.42% and an identity of 99.98%), two from different species but the same genus as one of this work (GenBank Accession No. CP022176.1, *Aeromonas salmonicida* S44, isolated from Atlantic salmon from the RAS Atlantic Salmon facility in China, with a coverage of 75.33% and an identity of 99.98%; GenBank Accession No. CP102110.1, *Aeromonas hydrophila* strain GD21SC2284TT, isolated from fish in China, with a coverage of 75.31% and an identity of 99.98%). Another two were from the genus *Enterobacter* of the family *Enterobacteriaceae* (GenBank Accession No. CP126807.1, *Enterobacter hormaechei*, isolated from cat urine in Switzerland, with a coverage of 75.31% and an identity of 99.98%; GenBank Accession No. CP126811.1, *Enterobacter hormaechei*, isolated from a swab of a dog’s hip joint in Switzerland, with a coverage of 75.29% and an identity of 99.98%). In contrast to these five sequences, the sequence of *A. caviae* QZ63 in this work carried an additional Tn-*tetA*-like fragment, and no Tn-*tetA*-like sequence was available in the NCBI nucleotide database.^2^

Next to the AAFT unit was an integron that carried five resistance gene, and the sequence sharing the highest similarity with it was from the plasmid P2 of *Klebsiella pneumoniae* 1,083 isolated from a patient at the Hospital Clinic (Barcelona), Spain (GenBank Accession No. OW967807.1, coverage 74.13% and identity 100%). Notably, even though there were integrons (or a truncated integron) next to the AAFT unit, such as fragments of the five sequences mentioned above, the gene from this work were completely different from any of the other five (Figure 5). The right side transposon (TnBB) of the 31.7 kb MDR region mainly consisted of a repeat sequence (*bla*_NDM-1_-*ble*-*tfrp*-*dsbD*-*hp*-*bla*_NDM-1_*-ble*-*tfrp*-*dsbD*-*hp*) flanked by two insert sequences (ISs), namely, IS*26* and IS*6100*. TnBB showed the highest similarity to a sequence from the *Escherichia coli* plasmid pLZ135-NDM isolated from a patient in Hong Kong, China (GenBank Accession No. MF353156.1, coverage 74.56% and identity 99.95%).

From the analysis above, it could be concluded that similar sequences of the 31.7 kb MDR region of *A. caviae* QZ63 could be found to be located on plasmids and/or in the chromosomes of bacteria of different species or genera, isolated from different sources (such as human beings, animals, fishes and the environment) worldwide. This indicated that the resistance genes carried by MGEs spread among bacteria of different species or genera by means of horizontal gene transfer, resulting in the worldwide dissemination of resistance.

### Comparative analysis of the extended-spectrum β-lactamase gene *bla*_PER-3_ in *Aeromonas hydrophila* QZ124

**Figure 6:**
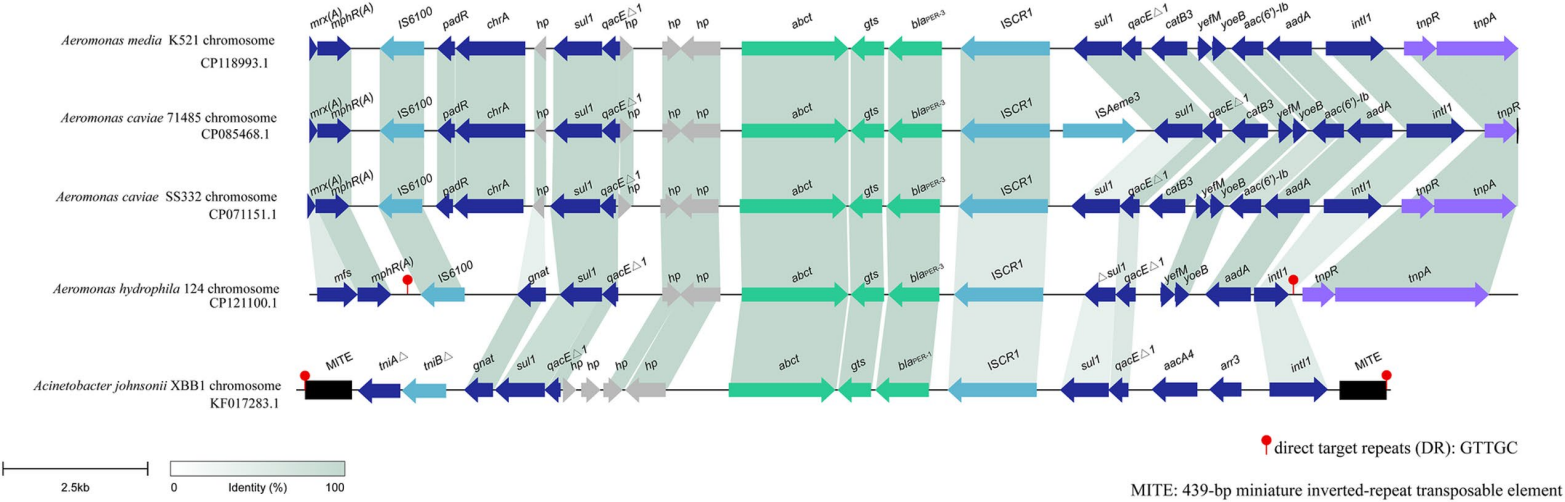
Synteny analysis of the 18 kbp region in the chromosome of *A. hydrophila* 124. hp, hypothetical protein. Accession numbers: *A. media* K521 chromosome (CP118993.1), *A. caviae* 71,485 chromosome (CP085468.1), *A. caviae* SS332 chromosome (CP071151.1), *Aeromonas caviae* GD21SC2284TT chromosome (AP021927.1), *Acinetobacter johnsonii* XBB1 chromosome (KF017283.1).

*bla*_PER-3_ is located in a variable region of the *A. hydrophila* QZ124 chromosome, and this region is approximately 18 kb in length and harbors several MGE-related sequences, including an intact integron carrying five resistance gene (*sul1*, *qacEΔ1*, *bla*_PER-3_, *Δsul1*, *qacEΔ1*, *aadA*), ISs (IS*6100* and IS*CR1*) and *tnp* genes (*tnpR* and *tnpA*). *bla*_PER-3_ was found immediately downstream an insertion sequence IS*CR1* (Figure 6). When searching for similar sequences in the nonredundant nucleotide database of NCBI, four sequences with greater similarity were retrieved. These sequences included one from *A. media* K521 (GenBank Accession No. CP118993.1, with a coverage of 83.29% and an identity of 99.93%), one from *A. caviae* 71,485 (GenBank Accession No. CP085468.1, with a coverage of 79.53% and an identity of 99.93%), one from *A. caviae* SS332 (GenBank Accession No. CP071151.1, with a coverage of 85.31% and an identity of 99.91%), and one from *Acinetobacter johnsonii* XBB1 (GenBank Accession No. KF017283.1, with a coverage of 72.76% and an identity of 99.92%) (Figure 6).

The bacteria harboring these four sequences were all isolated from human specimens in China. The three isolates with the highest similarities (CP118993.1, CP085468.1, and CP071151.1) to the sequence in this study were all from the same genus, *Aeromonas*, and were all isolated from the same province (Zhejiang) in China. The one (KF017283.1) with the lowest similarity was from a different bacterial family (*Acinetobacter johnsonii*) and was isolated from a different province (Sichuan) in China. This finding suggested that this *bla*_PER_-containing fragment may be carried mainly by *Aeromonas* strains and has also spread to bacteria with distant phylogenetic relationships in China. *bla*_PER_ has been found to be related to MGEs, which facilitate the mobility of this gene. The association of *bla*_PER_ with IS*Pa12* and Tn*1213* (a composite transposon composed of IS*Pa12* and its close relative IS*Pa13*) in *Pseudomonas aeruginosa* RNL-1 and *Klebsiella pneumoniae* CS1711 has been reported (Bae et al., 2011; Nordmann et al., 1993), and IS*CR1* with *bla*_PER_ was also found in *A. johnsonii* XBB1 and *A. baumannii* NF812784 (Wang et al., 2012; Zong, 2014). Several variants of Tn*1213* have also been identified, including IS*Pa12* fragmented by IS*6100* or IS*Ppu17* and IS*Prst1* inserted into Tn*1213* (Mantengoli and Rossolini, 2005).

### Time-kill assay

**Figure 7:**
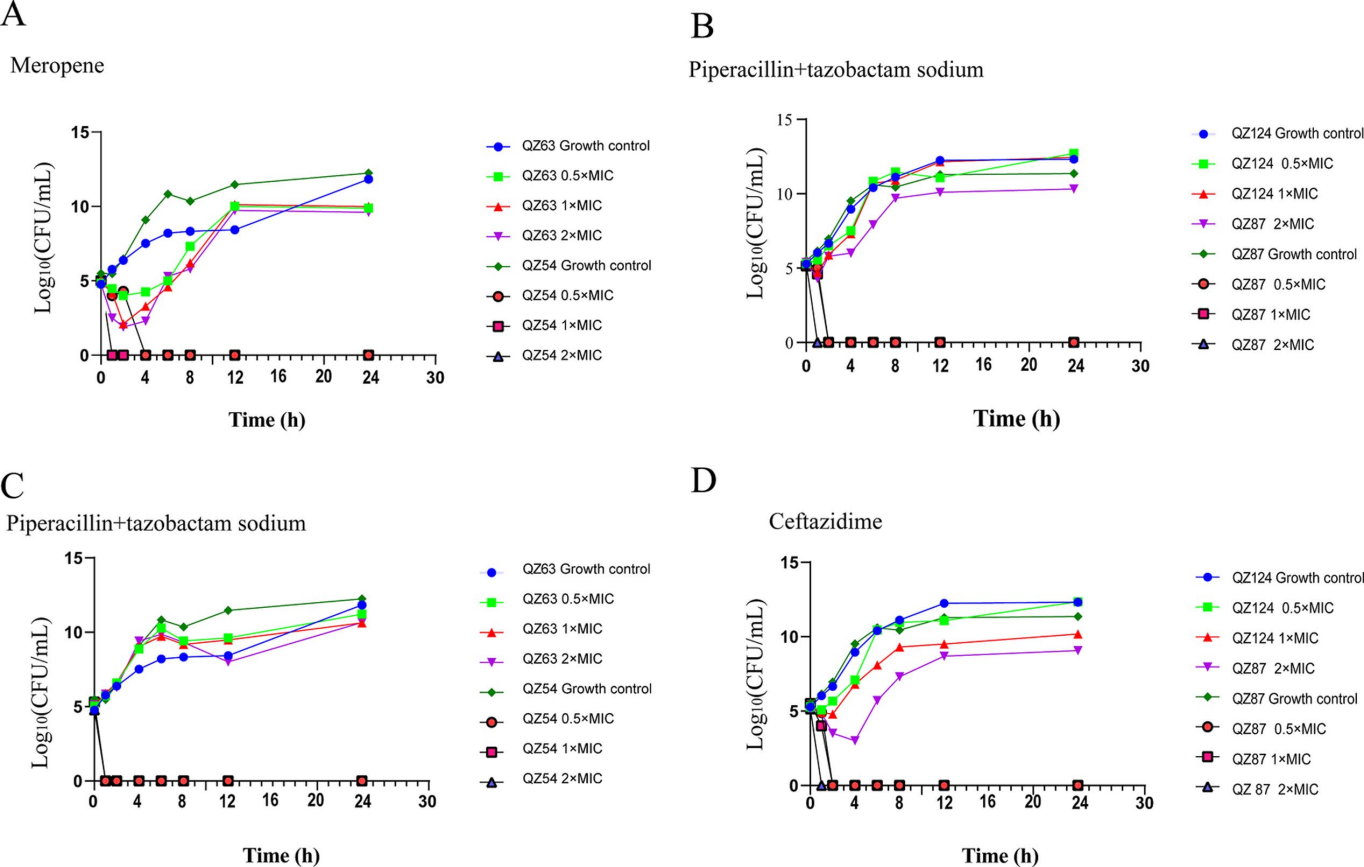
Time-kill curves showing the antibacterial effects. (A) meropenem (MIC: 2 µg/mL), (B,C) piperacillin + tazobactam (MIC: 32 µg/mL), and (D) ceftazidime (MIC: 32 µg/mL) at 0.5 × MIC, 1 × MIC, and 2 × MIC.

The results of time-kill assays demonstrated that *A. caviae* QZ63 harboring *bla*_NDM-1_ resumed growth 2 h after exposure to meropenem at 0.5 ×, 1 × and 2 × MIC. In contrast, the control strain QZ54 ceased growth within 1–2 h under meropenem concentrations of 0.5 ×, 1 ×, and 2 × MIC. The strain QZ124 (carrying the *bla*_PER-3_ gene) resumed growth after 1 h of exposure to ceftazidime at 0.5 × MIC. When treated with 1 × MIC, growth was delayed and resumed only after 2 h, whereas treatment with 2 × MIC resulted in a further delay, with growth resuming after 4 h. Conversely, the control strain QZ87 ceased growth within 1–2 h at all tested concentrations (0.5 ×, 1 ×, and 2 × MIC). The antimicrobial combination of piperacillin + tazobactam exhibited no inhibitory effects on QZ63 (harboring *bla*_NDM-1_) and QZ124 (harboring *bla*_PER-3_). In contrast, the control strains QZ54 and QZ87 ceased growth within 1–2 h across all tested concentrations (0.5 ×, 1 ×, and 2 × MIC) (Figure 7). We observed that piperacillin + tazobactam was unable to inhibit the activity of metallo-β-lactamases (MBL) and extended-spectrum beta-lactamase (ESBL), a phenomenon that has also been reported in other isolates producing NDM-1 (Davido et al., 2017). Therefore, monotherapy with piperacillin + tazobactam or similar agents should be avoided in infections caused by *A. caviae* harboring *bla*_NDM-1_. Moreover, the resistance genes *bla*_NDM-1_ and *bla*_PER-3_ in QZ63 and QZ124, respectively, were found to be associated with mobile genetic elements (MGEs), suggesting the potential for horizontal transfer. This warrants further attention.

## Conclusion

This study reported the correlation between the distribution of β-lactamase genes and the resistance phenotypes of clinical *Aeromonas* isolates, as well as the clinical characteristics of *Aeromonas* isolates. The increasing emergence of *Aeromonas* strains harboring extended-spectrum β-lactamase and metallo-β-lactamase genes poses a severe threat to public health. Close attention should be given to monitoring and controlling the spread of bacterial resistance.

## Data Availability

The original contributions presented in the study are publicly available. This data can be found here: https://www.ncbi.nlm.nih.gov/genbank/. The GenBank accession numbers for the chromosome and pQZ124-211 of A.hydrophila QZ124, and the chromosome of A. caviae QZ63, are CP121100, CP121101 and CP177201, respectively.

## Footnotes

^1^ http://pubmlst.org/ecloacae

^2^ https://www.ncbi.nlm.nih.gov/taxonomy
