# Supplementary material for: Whole-genome sequencing-based species classification, multilocus sequence typing, and antibiotic resistance mechanisms of the clinical Aeromonas complex
Source: Front Microbiol. 2025 Feb 25;16:1473150. doi: 10.3389/fmicb.2025.1473150 (PMC11893600; doi:10.3389/fmicb.2025.1473150)
Supplement: Supplementary file 1 [file Table_1.DOCX]

| Table S1. *Aeromonas* isolates used in this study | | | | | |
| --- | --- | --- | --- | --- | --- |
| Case no. | Age/sex | ANI(>95%) | MicroScan WalkAway system | ST | Source of specimen |
| 12 | 27/F | *A. caviae* | *A. enteropelogenes* | 1211-NEW | excrement |
| 30 | 61/M | *A. caviae* | *A. enteropelogenes* | 1213-NEW | excrement |
| 38 | 80/F | *A. caviae* | *A. caviae* | 181 | excrement |
| 46 | 66/F | *A. caviae* | *A. caviae* | 1221-NEW | excrement |
| 47 | 9months/M | *A. caviae* | *A. caviae* | 1222-NEW | excrement |
| 52 | 54/M | *A. caviae* | *A. caviae* | 1223-NEW | excrement |
| 53 | 17/M | *A. caviae* | *A. caviae* | 1223-NEW | excrement |
| 54 | 7months/M | *A. caviae* | *A. caviae* | 1224-NEW | excrement |
| 62 | 79/M | *A. caviae* | *A. caviae* | 928 | excrement |
| 63 | 82/M | *A. caviae* | *A. caviae* | 1225-NEW | excrement |
| 64 | 66/F | *A. caviae* | *A. caviae* | 1226-NEW | excrement |
| 69 | 93/M | *A. caviae* | *A. caviae* | 1229-NEW | excrement |
| 72 | 26/F | *A. caviae* | *A. caviae* | 1231-NEW | excrement |
| 82 | 44/F | *A. caviae* | *A. caviae* | 1235-NEW | excrement |
| 83 | 6/F | *A. caviae* | *A. caviae* | 1235-NEW | fester |
| 88 | 70/F | *A. caviae* | *A. hydrophila* | 1237-NEW | excrement |
| 89 | 18/M | *A. caviae* | *A. caviae* | 1238-NEW | excrement |
| 93 | 20/M | *A. caviae* | *A. hydrophila* | 1239-NEW | excrement |
| 95 | 63/F | *A. caviae* | *A. caviae* | 1319-NEW | bile |
| 96 | 55/F | *A. caviae* | *A. caviae* | 1363-NEW | excrement |
| 105 | 73/M | *A. caviae* | *A. caviae* | 1383-NEW | bile |
| 108 | 56/F | *A. caviae* | *A. caviae* | 1386-NEW | excrement |
| 114 | 72/M | *A. caviae* | *A. caviae* | 1388-NEW | urine |
| 117 | 62/F | *A. caviae* | *A. caviae* | 1388-NEW | urine |
| 122 | 79/M | *A. caviae* | *A. caviae* | 1388-NEW | urine |
| 123 | 88/F | *A. caviae* | *A. hydrophila* | 1392-NEW | bile |
| 137 | 65/M | *A. caviae* | *A. caviae* | 1399-NEW | whole blood |
| 9 | 49/M | *A. hydrophila* | *A. hydrophila* | 1209-NEW | fester |
| 15 | 84/M | *A. hydrophila* | *A. hydrophila* | 764 | bile |
| 16 | 50/M | *A. hydrophila* | *A. hydrophila* | 764 | fester |
| 22 | 54/M | *A. hydrophila* | *A. hydrophila* | 1212-NEW | fester |
| 56 | 86/M | *A. hydrophila* | *A. hydrophila* | 367 | whole blood |
| 67 | 56/F | *A. hydrophila* | *A. Veronii* | 1142 | fester |
| 73 | 55/M | *A. hydrophila* | *A. hydrophila* | 1232-NEW | sputum |
| 87 | 54/F | *A. hydrophila* | *A. hydrophila* | 1236-NEW | fester |
| 94 | 78/M | *A. hydrophila* | *A. hydrophila* | 1240-NEW | fester |
| 110 | 75/F | *A. hydrophila* | *A. hydrophila* | 1387-NEW | bile |
| 120 | 60/M | *A. hydrophila* | *A. hydrophila* | 1391-NEW | bile |
| 124 | 52/M | *A. hydrophila* | *A. hydrophila* | 1393-NEW | urine |
| 125 | 74/M | *A. hydrophila* | *A. hydrophila* | 1394-NEW | urine |
| 127 | 68/F | *A. hydrophila* | *A. hydrophila* | 1395-NEW | fester |
| 128 | 69/F | *A. hydrophila* | *A. hydrophila* | 1395-NEW | fester |
| 132 | 55/M | *A. hydrophila* | *A. Veronii* | 1397-NEW | sputum |
| 133 | 75/M | *A. hydrophila* | *A. hydrophila* | 721 | urine |
| 134 | 55/F | *A. hydrophila* | *A. hydrophila* | 1398-NEW | bile |
| 140 | 26/F | *A. hydrophila* | *A. Veronii* | 1401-NEW | bile |
| 141 | 64/M | *A. hydrophila* | *A. hydrophila* | 1401-NEW | fester |
| 19 | 67/M | *A. dhakensis* | *A. hydrophila* | 775 | bile |
| 31 | 37/M | *A. dhakensis* | *A. hydrophila* | 1214-NEW | excrement |
| 40 | 19/M | *A. dhakensis* | *A. hydrophila* | 788 | excrement |
| 74 | 76/M | *A. dhakensis* | *A. hydrophila* | 529 | excrement |
| 78 | 37/F | *A. dhakensis* | *A. jandaei* | 1234-NEW | excrement |
| 91 | 41/M | *A. dhakensis* | *A. hydrophila* | 518 | excrement |
| 106 | 82/M | *A. dhakensis* | *A. caviae* | 311 | sputum |
| 107 | 68/M | *A. dhakensis* | *A. jandaei* | 311 | whole blood |
| 119 | 33/M | *A. dhakensis* | *A. jandaei* | 1390-NEW | whole blood |
| 131 | 83/M | *A. dhakensis* | *A. hydrophila* | 710 | whole blood |
| 135 | 63/M | *A. dhakensis* | *A. hydrophila* | 534 | bile |
| 33 | 12/F | *A. enteropelogenes* | *A. enteropelogenes* | 1215-NEW | excrement |
| 39 | 41/M | *A. enteropelogenes* | *A. enteropelogenes* | 1217-NEW | excrement |
| 42 | 28/F | *A. enteropelogenes* | *A. enteropelogenes* | 1218-NEW | excrement |
| 100 | 53/F | *A. enteropelogenes* | *A. enteropelogenes* | 1368-NEW | excrement |
| 109 | 54/M | *A. enteropelogenes* | *A. enteropelogenes* | 930 | excrement |
| 10 | 57/M | *A. Veronii* | *A. Veronii* | 1210-NEW | sputum |
| 36 | 16/M | *A. Veronii* | *A. Veronii* | 1216-NEW | excrement |
| 44 | 14/M | *A. Veronii* | *A. Veronii* | 1221-NEW | excrement |
| 45 | 69/M | *A. Veronii* | *A. Veronii* | 1220-NEW | fester |
| 50 | 59/M | *A. Veronii* | *A. Veronii* | 464 | bile |
| 65 | 79/M | *A. Veronii* | *A. Veronii* | 1227-NEW | excrement |
| 68 | 15/M | *A. Veronii* | *A. hydrophila* | 1228-NEW | excrement |
| 75 | 48/M | *A. Veronii* | *A. Veronii* | 419 | excrement |
| 77 | 37/F | *A. Veronii* | *A. Veronii* | 1233-NEW | excrement |
| 79 | 27/M | *A. Veronii* | *A. caviae* | 512 | excrement |
| 138 | 58/M | *A. Veronii* | *A. Veronii* | 1400-NEW | fester |
| 139 | 88/M | *A. Veronii* | *A. Veronii* | 434 | bile |
| 104 | 72/M | *A. rivpollensis* | *A. Veronii* | 1386-NEW | bile |
| 111 | 88/M | *A. jandaei* | *A. jandaei* | 2218-NEW | fester |
| 130 | 67/M | New specie | *A. hydrophila* | 1396-NEW | whole blood |
