# Supplementary material for: Whole-genome sequencing-based species classification, multilocus sequence typing, and antibiotic resistance mechanisms of the clinical Aeromonas complex
Source: Front Microbiol. 2025 Feb 25;16:1473150. doi: 10.3389/fmicb.2025.1473150 (PMC11893600; doi:10.3389/fmicb.2025.1473150)
Supplement: Supplementary file 2 [file Table_2.docx]

**Table S2 | Distribution of β-lactam resistance genes**

| **Class** | **Genotype** | **Total(number)** | **Percentage of Class Genes** | **Species Distribution** |
| --- | --- | --- | --- | --- |
| A | *bla*_CTX-M_,  *bla*_TEM_,*bla*_RSA_,*bla*_PER_ | 10 | 5.30% | *A. caviae*  *A. hydrophila* |
| B | *bla*_IMP_，*cphA3*，*imiH*，*bla*_AFM_，*bla*_VIM_，*bla*_NDM_ | 50 | 26.70% | *A. caviae*  *A. hydrophila*  *A. dhakensis*  *A.veronii，*Others |
| C | *bla*_MOX_，*cepS*，*bla*_AQU_，*bla*_TRU_ | 38 | 20.40% | *A. caviae*  *A. hydrophila*  *A. dhakensis*  *A.enteropelogenes* |
| D | *bla*_OXA_ | 89 | 47.60% | *A. caviae*  *A. hydrophila*  *A. dhakensis*  *A.veronii，*Others |
